# The impact of socioeconomic deprivation on the risk of atrial fibrillation in patients with diabetes mellitus: A nationwide population-based study

**DOI:** 10.3389/fcvm.2022.1008340

**Published:** 2022-11-16

**Authors:** Minju Han, So-Ryoung Lee, Eue-Keun Choi, Sang-Hyeon Park, HuiJin Lee, Jaewook Chung, JungMin Choi, Kyung-Do Han, Seil Oh, Gregory Y. H. Lip

**Affiliations:** ^1^Department of Internal Medicine, Seoul National University Hospital, Seoul, South Korea; ^2^Department of Internal Medicine, Seoul National University College of Medicine, Seoul, South Korea; ^3^Statistics and Actuarial Science, Soongsil University, Seoul, South Korea; ^4^Liverpool Centre for Cardiovascular Science, Liverpool Heart and Chest Hospital, University of Liverpool, Liverpool, United Kingdom; ^5^Department of Clinical Medicine, Aalborg University, Aalborg, Denmark

**Keywords:** socioeconomic status, atrial fibrillation, social medicine, diabetes mellitus, medical aid beneficiaries

## Abstract

**Objective:**

To evaluate the relationship between socioeconomic status and the risk of atrial fibrillation (AF) in patients with diabetes mellitus (DM).

**Research design and methods:**

From the National Health Insurance Service (NHIS) database, we identified 2,429,610 diabetic patients who underwent national health check-ups between 2009 and 2012. Tracing back the subjects for 5 years from the date of health check-up, we determined the subjects’ income and whether they received medical aid (MA) during the past 5 years. Subjects were divided into six groups according to the number of years of receiving (MA groups 0 through 5) and into four groups according to socioeconomic status change during the past 5 years. We estimated the risk of AF for each group using the Cox proportional-hazards model.

**Results:**

During a median follow-up of 7.2 ± 1.7 years, 80,257 were newly identified as AF. The MA groups showed a higher risk of AF than the non-MA group with the hazard ratios (HRs) and 95% confidence interval (CI) 1.32 (1.2–1.44), 1.33 (1.22–1.45), 1.23 (1.13–1.34), 1.28 (1.16–1.4), and 1.50 (1.39–1.63) for MA groups 1 through 5, respectively. Dividing subjects according to socioeconomic condition change, those who experienced worsening socioeconomic status (non-MA to MA) showed higher risk compared to the persistent non-MA group (HR 1.54; 95% CI 1.38–1.73).

**Conclusion:**

Low socioeconomic status was associated with the risk of AF in patients with diabetes. More attention should be directed at alleviating health inequalities, targeting individuals with socioeconomic deprivation to provide timely management for AF.

## Introduction

The prevalence of atrial fibrillation (AF) and AF-related morbidity and mortality is increasing globally. It is associated with an increasingly older adult population, more prevalent comorbidities, and unhealthy lifestyles (1, 2). For example, the Framingham study identified aging and cardiovascular comorbidities such as hypertension, diabetes mellitus (DM), coronary artery disease, and valvular heart disease as the most potential risk factors for the development of AF (3).

Diabetes mellitus is known to increase the risk of AF by 28% (4, 5) and is an important factor in the prognosis of AF. In addition, the risk of AF is increased in patients with poorly controlled DM and macrovascular and microvascular complications (6). Prevention of incident AF and appropriate management of the prevalent AF in patients with diabetes would lower the risk of morbidity and mortality. Indeed, this holistic or integrated care approach to AF care is being increasingly promoted (7, 8), given that adherence to holistic management is associated with improved clinical outcomes (9, 10).

Among the non-disease risk factors for AF, many studies have been conducted on whether the risk of AF changes according to health inequalities and socioeconomic status (11–16). Although socioeconomic status shows an inverse relationship with overall morbidity, mortality, and cardiovascular diseases (17, 18), the results on the relationship between socioeconomic status and AF remain controversial. Previous studies have shown that a lower income is associated with a higher risk of AF (11, 12). However, a weak relationship between a low socioeconomic status and the risk of AF has also been reported (13). Nevertheless, low socioeconomic status notably elevates the prevalence of various diseases, including DM, obesity, and depression (19, 20), and these diseases are risk factors for AF (3, 21).

We investigated the association between socioeconomic status and AF risk in patients with diabetes using a population-based nationwide cohort study.

## Materials and methods

### Data source and study population

We used data from the National Health Insurance Service (NHIS) database. The Korean NHIS is a compulsory public medical assistance system with over 51 million Koreans currently participating. In addition, the Korean National Health Insurance Corporation provides annual or biennial national health examinations for people over the age of 20, and these data are linked to the NHIS database.

**FIGURE 1:**
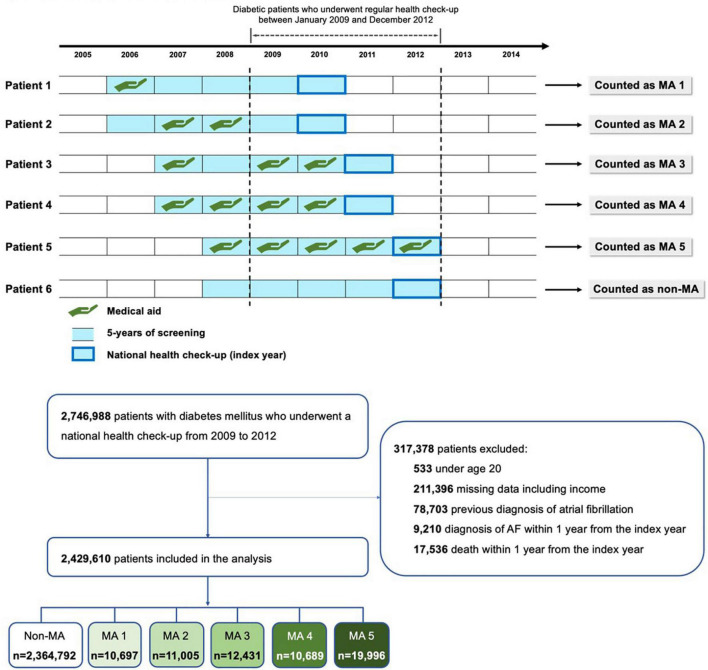
Study design and flowchart of enrolment.

We identified 2,429,610 subjects with DM and without prevalent AF, who underwent a national health examination at least once between 2009 and 2012. Patients younger than 20 years of age, those with missing values among covariates, and those diagnosed with AF within 1 year after the health examination were excluded. Figure 1 shows the flowchart of study enrolment. This study was exempt from review by the Seoul National University Hospital Institutional Review Board (E-2105-141-1220).

### Definition of medical aid beneficiary

Medical aid (MA) is a public assistance system that the state guarantees for the medical problems of low-income people who cannot sustain life or have difficulties in living (22). People who are unable to work and homeless are usually recipients of the MA; the recipient household’s income must be less than 40% of the median national household income to benefit from the MA (23).

By tracing back 5 years from the index date of the subject’s health examination, we obtained the participants’ household income status and categorized the study population into six groups according to the number of years the subject was a beneficiary of the MA program (0 through 5; named non-MA, MA 1, MA 2, MA 3, MA 4, and MA 5 groups, respectively). The detailed study design is illustrated in Figure 1.

### Assessment of covariates

Covariates included age, sex, smoking status, drinking status, regular exercise, comorbidities of hypertension and dyslipidaemia, body mass index (BMI), waist circumference, systolic blood pressure (SBP), diastolic blood pressure (DBP), fasting blood glucose level, total cholesterol, and high and low-density lipoprotein (HDL and LDL) cholesterol (24). The details of the subjects’ DM were analyzed to determine whether they had been diagnosed with DM 5 years or more, whether the subject was taking DM medication, and whether the number of diabetic medications was three or more (25). Smoking status (never, ex, or current smoker), drinking status, and physical activity were assessed using a self-report questionnaire completed as part of the national health examination (26). Alcohol consumption of less than 30 g per day was defined as mild drinking, and 30 g or more was defined as heavy drinking. Regular physical activity was defined as performing moderate-intensity exercise more than five times a week or vigorous-intensity exercise more than three times a week (26). Detailed definitions of the diagnoses, including AF and comorbidities, such as hypertension and dyslipidaemia, are presented in Supplementary Table 1 (24).

### Study outcome and follow-up

The primary outcome was the occurrence of incident AF during the follow-up period. AF was defined as at least one hospitalization or at least two outpatient clinic visits with diagnostic codes of AF (I480-I484 and I489), according to the International Classification of Disease, 10th Revision (ICD-10) (6, 27, 28). Subjects were followed up from the index date of the national health examination until the occurrence of AF, death, or the end of the study period (31 December 2018), whichever came first.

### Statistical analysis

Baseline characteristics were described across groups with different numbers of years of receiving MA. Continuous variables were expressed as mean ± standard deviation, and categorical variables were presented as numbers and percentages. Differences among the groups were examined using analysis of variance (ANOVA). The crude incidence rate (IR) of incident AF was calculated as the number of events per 1,000 person-years (PY). To analyze the association between the number of years of receiving MA and the risk of incident AF, we used univariate and multivariate Cox proportional hazard regression models. The outcomes for the groups were presented as hazard ratios (HRs) and 95% confidence intervals (CIs). Adjustments were made for the covariates of age, sex, hypertension, dyslipidaemia, BMI, fasting blood glucose level, smoking, drinking status, and regular physical activity.

The level of significance was set at 0.05 and all analyses were two-sided. Statistical analyses were conducted using SAS version 9.4 (SAS Institute, Cary, NC, USA).

### Complementary analyses

We further conducted multiple complementary analyses to investigate the associations between various indicators of SES and the risk of incident AF. First, to check whether there is a relationship between various income levels and AF risk, participants were divided into 21 groups according to health insurance premiums paid in the index year (the year of national health examination): the MA group and income level 1 through 20 groups, with higher numbers indicating higher insurance premiums. We cross-sectionally analyzed the effect of income level on the risk of AF in the index year. Second, to check whether low income–applying a more lenient definition than MA beneficiary–defined as paying the bottom 20% of health insurance premiums also affects the risk of AF, we divided the participants into six groups according to the number of years of low income. Lastly, to check whether changes in socioeconomic status affect AF risk, whether the subjects received MA in the index year and 4 years ago from the index year were investigated. Subjects were classified into four groups: initial MA recipients and MA recipients later (persistent MA group), initial non-MA recipients but later becoming MA recipients (non-MA to MA group), initial MA recipients but later becoming non-MA recipients (MA to non-MA group), and initial non-MA recipients and non-MA recipients later (persistent non-MA group). In these three complementary analyses, the multivariable-adjusted HRs of incident AF among the groups were estimated.

### Subgroup analyses

We performed subgroup analyses and interaction tests to evaluate the potential impact of age, sex, duration of DM, insulin use, use of three or more antidiabetic medications, and comorbidities, including hypertension and dyslipidaemia, on the relationship between socioeconomic status and the risk of AF. P for interaction less than 0.1 was considered significant.

## Results

Among a total of 2,429,610 subjects (mean age 56.9 ± 12.4, 60% men), 2,364,792 did not have a history of MA (97.3%, non-MA group), and 10,697 subjects had a history of MA at least for 1 year (0.4%, MA 1 group), 11,005 for 2 years (0.5%, MA 2 group), 12,431 for 3 years (0.5%, MA 3 group), 10,689 for 4 years (0.4%, MA 4 group), and 19,996 subjects benefited from MA for 5 years (0.8%, MA 5 group) (Figure 1). Table 1 shows the baseline characteristics of each group.

**TABLE 1:** Baseline characteristics of the subjects grouped by the number of times receiving medical aid.

	Cumulative medical aid burden						
	0	1	2	3	4	5	*P*-value

	*n* = 2,364,792	*n* = 10,697	*n* = 11,005	*n* = 12,431	*n* = 10,689	*n* = 19,997	
Age	56.82 ± 12.44	58.5 ± 13.18	59.82 ± 13.11	60.52 ± 12.39	60.1 ± 12.41	56.49 ± 8.73	<0.0001
<40	199,126 (8.42%)	705 (6.59%)	545 (4.95%)	417 (3.35%)	376 (3.52%)	326 (1.63%)	<0.0001
40–64	1,486,221 (62.85%)	6,042 (56.48%)	6,091 (55.35%)	7,022 (56.49%)	6,049 (56.59%)	14,616 (73.09%)	
≥65	679,445 (28.73%)	3,950 (36.93%)	4,369 (39.7%)	4,992 (40.16%)	4,264 (39.89%)	5,054 (25.28%)	
Male sex	1,429,887 (60.47%)	4,938 (46.16%)	4,642 (42.18%)	5,068 (40.77%)	4,341 (40.61%)	9,195 (45.98%)	<0.0001
Smoking							<0.0001
Never	1,305,493 (55.21%)	6,633 (62.01%)	7,133 (64.82%)	8,220 (66.13%)	7,104 (66.46%)	11,920 (59.61%)	
Former	428,469 (18.12%)	1,260 (11.78%)	1,250 (11.36%)	1,355 (10.9%)	1,106 (10.35%)	2,332 (11.66%)	
Current	630,830 (26.68%)	2,804 (26.21%)	2,622 (23.83%)	2,856 (22.97%)	2,479 (23.19%)	5,744 (28.73%)	
Drinking							
Non-MA	1,333,833 (56.4%)	7,390 (69.08%)	7,965 (72.38%)	9,132 (73.46%)	7,866 (73.59%)	14,878 (74.4%)	
Mild	787,339 (33.29%)	2,464 (23.03%)	2,341 (21.27%)	2,476 (19.92%)	2,158 (20.19%)	3,813 (19.07%)	
Heavy	243,620 (10.3%)	843 (7.88%)	699 (6.35%)	823 (6.62%)	665 (6.22%)	1,305 (6.53%)	
Regular exercise	488,724 (20.67%)	1,645 (15.38%)	1,706 (15.5%)	1,922 (15.46%)	1,627 (15.22%)	3,159 (15.8%)	<0.0001
Hypertension	1,295,148 (54.77%)	6,502 (60.78%)	7,033 (63.91%)	8,184 (65.84%)	6,970 (65.21%)	12,848 (64.25%)	<0.0001
Dyslipidemia	935,703 (39.57%)	4,720 (44.12%)	5,081 (46.17%)	5,980 (48.11%)	5,306 (49.64%)	10,978 (54.9%)	<0.0001
BMI, kg/m^2^	25.06 ± 3.88	24.92 ± 3.79	24.96 ± 3.88	25.01 ± 3.87	24.98 ± 3.91	25.29 ± 4.13	<0.0001
BMI < 18.5	36,462 (1.54%)	323 (3.02%)	309 (2.81%)	354 (2.85%)	303 (2.83%)	616 (3.08%)	
18.5 ≤ BMI < 23	588,690 (24.89%)	2,941 (27.49%)	3,124 (28.39%)	3,441 (27.68%)	3,039 (28.43%)	5,231 (26.16%)	
23 ≤ BMI < 25	589,915 (24.95%)	2,442 (22.83%)	2,383 (21.65%)	2,758 (22.19%)	2,295 (21.47%)	4,119 (20.6%)	
25 ≤ BMI < 30	917,367 (41.08%)	4,001 (37.4%)	4,147 (37.68%)	4,667 (37.54%)	4,023 (37.64%)	7,582 (37.92%)	
30 ≤ BMI	178,358 (7.54%)	990 (9.25%)	1,042 (9.47%)	1,211 (9.74%)	1,029 (9.63%)	2,448 (12.24%)	
Waist circumference, cm	85.34 ± 8.88	84.93 ± 9.43	85.16 ± 12	85.1 ± 9.53	85.02 ± 12.14	85.85 ± 10.03	<0.0001
SBP, mmHg	129.01 ± 15.87	128.42 ± 16.65	128.51 ± 16.57	128.59 ± 16.76	128.26 ± 16.57	126.42 ± 16.37	<0.0001
DBP, mmHg	79.11 ± 10.29	78.49 ± 10.49	78.32 ± 10.33	78.38 ± 10.4	78.2 ± 10.4	77.73 ± 10.42	<0.0001
Glucose, mg/dL	137.98 ± 47.89	141.09 ± 54.27	141.33 ± 54.79	140.62 ± 54.06	141.22 ± 55.33	141.23 ± 54.78	<0.0001
Total cholesterol, mg/dL	197.53 ± 46.02	195.32 ± 44.51	193.52 ± 49.97	192.12 ± 45.62	192.77 ± 49.37	187.7 ± 44.84	<0.0001
HDL, mg/dL	52.49 ± 31.91	52.46 ± 28.36	52.01 ± 25.33	52.03 ± 47.87	51.99 ± 24.56	51.21 ± 39.87	<0.0001
LDL, mg/dL	113.81 ± 92.14	111.24 ± 63.33	110.58 ± 62.65	110.49 ± 132.27	108.6 ± 51.75	104.61 ± 43.59	<0.0001
Diabetes mellitus							<0.0001
Disease duration ≥5 year	706,448 (29.87%)	3,522 (32.93%)	4,140 (37.62%)	5,021 (40.39%)	3,787 (35.43%)	9,069 (45.35%)	<0.0001
OnDM medication	1,280,126 (54.13%)	7,054 (65.94%)	7,661 (69.61%)	9,240 (74.33%)	7,835 (73.3%)	16,345 (81.74%)	<0.0001
≥ 3DM medications	323,077 (13.66%)	1,988 (18.58%)	2,211 (20.09%)	2,762 (22.22%)	2,393 (22.39%)	4,931 (24.66%)	<0.0001

Compared with the non-MA group, the MA group had a higher prevalence of hypertension and dyslipidaemia and a lower prevalence of regular physical activity. The proportion of obese people with BMI ≥ 25 kg/m^2^ was significantly higher in the MA 5 group (48.6% in the non-MA group, 46.6% in the MA 1, 47.2% in the MA 2 group, 47.3% in the MA 3 group, 47.3% in the MA 4 group, and 50.2% in the MA 5 group; *p* < 0.0001). The proportion of patients with a DM duration of 5 years or longer was significantly higher in the MA group than in the non-MA group (29.9 vs. 32.9 to 45.4% in the MA ≥ 1 group, *p* < 0.0001). The proportion of subjects taking three or more antidiabetic drugs was higher in the MA group than in the non-MA group (18.6 to 24.7% in the MA group vs. 13.7% in the non-MA group, *p* < 0.0001).

The baseline characteristics based on the occurrence of AF during the follow-up period are summarized in Supplementary Table 2. Patients who developed new AF during follow-up (AF group) were older than those who did not develop the arrhythmia (non-AF group) (65.1 ± 10.32 years in AF group vs. 56.59 ± 12.39 years in non-AF group, *p* < 0.0001), while the sex ratio between groups was similar (men 59.93% in AF group vs. 60.02% in non-AF group, *p* = 0.6272). The incidence of underlying hypertension and dyslipidaemia was higher in the AF group (hypertension 72.34% in AF group vs. 54.42%, *p* < 0.0001; dyslipidaemia 42.21% in AF group vs. 39.75%, *p* < 0.0001). Finally, the proportion of patients who were diagnosed with diabetes more than 5 years ago and who used three or more antidiabetics was also higher in the AF group (duration of diabetes ≥5 years 39.31% in the AF group vs. 29.81% in the non-AF group, *p* < 0.0001; ≥three antidiabetics 16.52% in the AF group vs. 13.8% in the non-AF group, *p* < 0.0001).

### The number of years of receiving medical aid and the risk of incident atrial fibrillation

**FIGURE 2:**
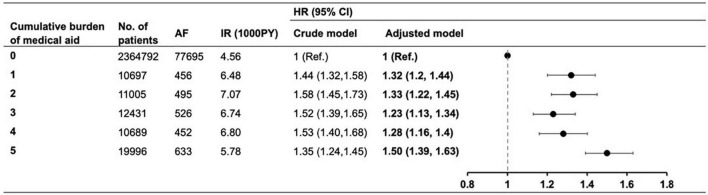
The risk of atrial fibrillation according to the cumulative burden of medical aid. Adjusted model corrected for age, sex, body mass index, blood glucose level, smoking, drinking, regular physical activity, hypertension, dyslipidemia, insulin use, ≥3 antidiabetic medication, and ≥5 years of diabetes mellitus duration. AF, atrial fibrillation; IR, incidence rate; PY, person-year; HR, hazard ratio; CI, confidence interval.

During the mean of 7.2 ± 1.7 years of follow-up (17,436,758 PY), AF was newly diagnosed in 80,257 patients (3.30%). The crude IR and the unadjusted and adjusted HRs for each group are summarized in Figure 2. The risk of AF was higher by 23% to 50% in the MA groups than in the non-MA group: the adjusted HRs (95% CI) in the MA 1 group 1.32 (1.20–1.44); 1.33 (1.22–1.45) in the MA 2 group; 1.23 (1.13–1.34) in the MA 3 group; 1.28 (1.16–1.4) in the MA 4 group, and 1.50 (1.39–1.63) in the MA 5 group. Notably, the MA 5 group showed the highest risk of AF compared to the non-MA group.

### Income levels at the index year and the risk of incident atrial fibrillation

To understand the relationship between income level and the risk of AF, we divided the participants who did not receive MA as of the index year (the year of the national health examination) into 20 groups according to their income level. The income level was estimated using the amount of health insurance premiums paid. We analyzed the risk of AF among 21 groups, comprising the MA beneficiary group (MA group) and income level 1–20 groups.

Compared with group 20, the group of subjects estimated to have the highest income, the adjusted HRs of groups 1 to 19 showed an increasing trend of AF risk, whereas the MA group showed a 57% higher risk of AF (adjusted HR 1.57, 95% CI, 1.47–1.68, Supplementary Figure 1).

### The number of years with low income and the risk of incident atrial fibrillation

To check whether low income, which is defined as patients with income levels of less than 20% of the entire Korean population, also affects the risk of AF, we divided the total population into six groups in the same way according to the number of years of low income (Supplementary Figure 2). This applies a more lenient definition than that of the MA beneficiary criterion.

Patients with low income for 2, 3, 4, and 5 years were associated with a higher risk of AF compared to subjects without low income for 5 years: the adjusted HRs (95% CI) in patients with low income for 2 years 1.04 (1.01–1.07), for 3 years 1.07 (1.03–1.10), for 4 years 1.04 (1.01–1.08), and for 5 years 1.09 (1.06–1.12), whereas those with low income for 1 year did not show a significant difference. The association between the number of years with low income and incident AF was significantly attenuated compared with the association between the number of years with MA, and the risk of AF.

### Changes of socioeconomic status assessed with or without receiving medical aid and the risk of atrial fibrillation

**FIGURE 3:**
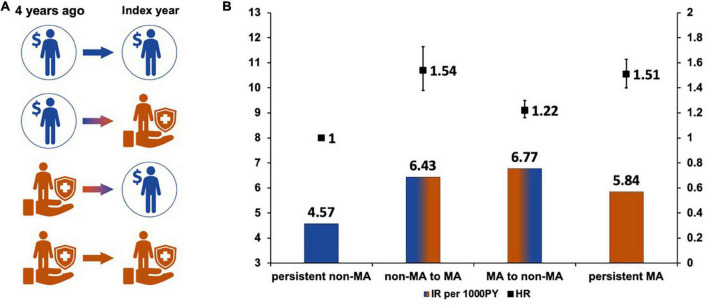
Changes in socioeconomic status and the risk of atrial fibrillation. **(A)** Changes of socioeconomic status. **(B)** Hazard ratios according to the changes of socioeconomic status.

To determine whether changes in socioeconomic status affected the risk of AF, we investigated whether the subject received MA in the index year and 4 years before the index year (Figure 3). Subjects who were not MA beneficiaries both in the index year and 4 years before the index year (persistent non-MA group) were regarded as the reference group.

The AF risk was higher in subjects who became the new MA group in the index year or who continuously belonged to MA groups (non-MA to MA group, adjusted HR 1.54, 95% CI 1.38–1.73; and persistent MA group 1.51, 95% CI 1.40–1.63). Subjects in the non-MA group at index year (MA to non-MA group) still had a 20% higher AF risk than those who were persistently in the non-MA group (adjusted HR 1.22, 95% CI 1.16–1.30).

### Subgroup analyses

Subgroup analyses were performed for age, sex, diabetes duration, insulin use, three or more antidiabetic drugs, hypertension, and dyslipidaemia (Supplementary Table 3). There was no significant interaction between the subgroups for each item, except for age (*p* for interaction <0.001). Among the age groups classified as <40, 40–64, ≥65 years, the 40–64-year age group and ≥65-year group showed the same trend as the main result, whereas the group under 40-year-old of age did not show an association between the risk of AF and the number of MA history. According to MA, the increase in AF risk was slightly attenuated in those aged ≥65 years and was most pronounced in the 40–64-year-old group.

## Discussion

In this study, we investigated the effect of SES on the risk of incident AF. The main findings of the study were as follows: (1) the MA group showed a 23–50% higher risk of AF compared to the non-MA group; (2) the cumulative burden of MA exposure up to five times showed the highest risk of AF compared to the non-MA group; (3) the risk of incident AF increased by 1.57 times in the MA group compared to the group with the highest income; and (4) those who became non-MA group during follow-up still had an increased risk of AF compared to the non-MA group, suggesting a legacy effect of MA on the risk of AF.

To the best of our knowledge, this is the first study to report the impact of the cumulative burden of MA exposure on AF risk, especially in patients with diabetes. Our study supports the alleviation of health inequalities by targeting individuals with socioeconomic deprivation in order to provide timely management of AF.

Diabetes is associated with worsening of AF prognosis and is a potent risk factor for AF (29, 30). Patients with AF and underlying DM also showed a higher risk of stroke than those without diabetes (29, 30). Thus, the diagnosis and appropriate management of AF in patients with diabetes are important for improving clinical outcomes. Health inequalities and socioeconomic status are also important factors influencing the outcome of patients with diabetes. Socioeconomic status has been associated with knowledge of diabetes, self-care, and clinical outcomes in patients with type 2 DM (31). In particular, diabetes outcomes comprising multiple components, such as HbA1c level, LDL level, blood pressure, and the physical and mental components of the QOL score, are correlated with socioeconomic factors (32–35). In our study, we further investigated the association between SES and the risk of AF in patients with diabetes and found a strong correlation between low SES and the risk of AF.

The literature shows that many studies on SES and various cardiovascular outcomes have shown an inverse correlation. A previous study reported an inverse relationship between socioeconomic status and almost all risk factors for CVD, including diabetes, obesity, smoking, and physical activity (36). More recently, in a population-based cohort study using data from the US National Health and Nutrition Examination Survey (US NHANES) and UK Biobank, adults with low socioeconomic status and no healthy lifestyle factors showed a higher risk of all-cause mortality and incident CVD higher (3.53 times and 2.09 times, respectively) than those with high socioeconomic status and healthy lifestyle factors (37). This study also reported that the influence of unhealthy lifestyle was smaller than that of socioeconomic inequity.

However, regarding the association between socioeconomic status and AF risk, previous studies have reported controversial results (11, 12, 38–40). Lower family income was associated with a higher risk of AF (11). Residents of lower socioeconomic status also had a higher risk of incident AF (12) and higher mortality when hospitalized for AF (38). Regarding studies that reported conflicting results, AF-related mortality was higher in European countries with higher GDP (39). The inverse relationship between socioeconomic status and AF risk was not evident in older adult individuals with the highest prevalence of AF (40).

Our study defined the cumulative burden of MA, enabling us to longitudinally identify the subjects’ socioeconomic status in the previous 5 years. Our study, which more comprehensively determines the subjects’ socioeconomic status using the concept of cumulative MA burden, confirms once again that there is an inverse correlation between low socioeconomic status and the risk of AF.

Becoming a beneficiary of MA is accepting the status of the socially underprivileged. The stigmatization of welfare beneficiaries has been studied sociologically for decades. Previous studies have reported an association between living on welfare, increased mental stress, and negative emotions (33, 34). The problem of poor self-care with health in people of low socioeconomic status is also a frequently studied topic (31, 41). The individual’s psychological stress and neglect of healthcare accompanying the process of accepting a new status as underprivileged might have increased the risk of AF. In addition, we found that an unhealthy lifestyle was more prevalent in the MA group than in the non-MA group. Patients in the MA group had a higher prevalence of current smoking and non-regular exercise than those in the non-MA group. This is consistent with the study results that socioeconomic inequity in various health outcomes is highly associated with lifestyle factors, such as smoking, alcohol consumption, physical activity, and diet (42–44). A previous study reported that smoking, alcohol use, and physical activity were significantly associated with new-onset AF (42). These findings imply that lifestyle factors may have a therapeutic value in patients with diabetes. For example, abstinence from alcohol is associated with a lower risk of developing AF in patients with newly diagnosed T2DM (43). In patients with newly diagnosed AF, current alcohol intake is associated with an increased risk of ischemic stroke, whereas alcohol abstinence after AF diagnosis is associated with a lower risk of ischemic stroke (44).

Interestingly, we found that AF risk differed according to socioeconomic status change. First, the risk of AF was similarly higher in those who received MA at the index year, regardless of a previous history of MA. Current SES had a more significant impact on the risk of AF than past SES. A worsening socioeconomic status would have resulted in greater psychological pressure on the subjects, increasing the risk of AF. Second, patients in the non-MA group at the index year showed different risks of AF according to their previous socioeconomic status. The group of patients who experienced an improvement in socioeconomic status (MA to non-MA group) still showed a 22% higher AF risk than the persistent non-MA group. This suggests a prophylactic effect of MA on the risk of AF, emphasizing that SES still has a considerable impact on clinical outcomes in patients with diabetes.

## Strengths and limitations

In our study, the socioeconomic status of the subjects was identified longitudinally by screening for 5 years, not at any single time point. This method has the advantage of being able to grasp the burden of the low socioeconomic status experienced by the subjects during the period, and further being able to determine whether there was a change in the socioeconomic status during the period, so that the situation of the subjects could be evaluated in a more diversified way. In addition, we limited the subjects of this study to patients with diabetes. Since DM is one of the notable risk factors for AF, setting this risk factor as the subject’s prerequisite allowed us to focus more on the influence of socioeconomic status. In addition, one of the strengths of our study is that we identified the socioeconomic status of the participants using the NHIS database (24). Instead of collecting information on income through self-questionnaires reported by participants, we improved the reliability of the results by using a database that records the exact amount of health insurance premium payments. The NHIS database holds information on all citizens residing in Korea in all age groups, reducing the possibility of selection bias, and the resident registration number jointly recorded further ensures the accuracy of information.

This study had some limitations. As mentioned, the subjects were limited to patients with diabetes at enrolment to focus more on the impact of socioeconomic status on incident AF. Therefore, this study alone cannot explain whether the results can be equally applied to the non-diabetic population. However, since all subjects had diabetes, the correction for diabetes was more reliable than when the entire population was enrolled. We believe that even when conducting further research on whether socioeconomic status affects the increase in AF risk among non-diabetic individuals, only non-diabetics should be included as subjects, since DM is a clear risk factor for AF (3, 5, 34) and the correction of its impact might not be complete in the coexistence of subjects with diabetes and those without diabetes in the analysis.

Low socioeconomic status is associated with the risk of AF in patients with diabetes. More attention should be directed at alleviating health inequalities and targeting individuals with socioeconomic deprivation to provide timely management of AF.

## Data availability statement

This study used publicly available datasets from Korean National Health Insurance Service Database https://nhiss.nhis.or.kr/bd/ab/bdaba021eng.do.

## Ethics statement

This study was exempt from review by the Seoul National University Hospital Institutional Review Board (E- 2105-141-1220). Written informed consent was not required for this study in accordance with the local legislation and institutional requirements.

## Author contributions

MH, S-RL, E-KC, SO, and GL initially conceptualized the subject of this study. S-RL and E-KC designed the study. MH, S-HP, HL, JC, and JMC performed the literature research and data collection. MH, S-RL, and K-DH analyzed the data. MH and S-RL were major contributors on writing the manuscript. All authors read and approved the final manuscript.
