# Supplementary material for: The impact of socioeconomic deprivation on the risk of atrial fibrillation in patients with diabetes mellitus: A nationwide population-based study
Source: Front Cardiovasc Med. 2022 Nov 16;9:1008340. doi: 10.3389/fcvm.2022.1008340 (PMC9708742; doi:10.3389/fcvm.2022.1008340)

**The impact of socioeconomic deprivation on the risk of atrial fibrillation in patients with diabetes mellitus: a nationwide population-based study**

Minju Han^1†^, So-Ryoung Lee^1†^, Eue-Keun Choi^1,2^, Sang-Hyeon Park^1^, HuiJin Lee^1^, Jaewook Chung^1^, JungMin Choi^1^, Kyung-Do Han^3^, Seil Oh^1,2^, Gregory Y.H. Lip^1,2,4,5^

^1^ Department of Internal Medicine, Seoul National University Hospital, Seoul, Republic of Korea

^2^ Department of Internal Medicine, Seoul National University College of Medicine, Seoul, Republic of Korea

^3^ Statistics and Actuarial Science, Soongsil University, Seoul, Republic of Korea

^4^ Liverpool Centre for Cardiovascular Science, University of Liverpool and Liverpool Heart and Chest Hospital, Liverpool, United Kingdom

^5^ Department of Clinical Medicine, Aalborg University, Aalborg, Denmark

† These authors contributed equally.

**Supplementary Materials**

**Supplementary Tables 1-3**

**Supplementary Figures 1-2**

**Supplementary Table 1. Definitions of covariates and clinical outcome**

| **Diagnosis** | **ICD-10-CM code and definition** | **Diagnostic definition** |
| --- | --- | --- |
| **Clinical outcome** |  |  |
| **Atrial fibrillation** | I48.0-48.4, I48.9 | Diagnostic code ≥1 during admission or ≥2 in outpatient department |
| **Comorbidities** |  |  |
| **Hypertension** | I10-I13, I15; and minimum 1 prescription of anti-hypertensive drug (thiazide, loop diuretics, aldosterone antagonist, alpha-/beta-blocker, calcium-channel blocker, angiotensin-converting enzyme inhibitor, angiotensin II receptor blocker). | Diagnostic code ≥1 during admission or ≥2 in outpatient department |
| **Diabetes mellitus** | E11-E14; and minimum 1 prescription of anti-diabetic drugs (sulfonylureas, metformin, meglitinides, thiazolidinediones, dipeptidyl peptidase-4 inhibitors, α-glucosidase inhibitors, and insulin). | Diagnostic code ≥1 during admission or ≥2 in outpatient department |
| **Dyslipidemia** | E78 | Diagnostic code ≥1 during admission or in outpatient department |

**Supplementary Table 2. Baseline characteristics of the subjects grouped by the occurrence of AF in the follow-up period**

|  | **Total** | **No AF** | **AF** | **P-value** |
| --- | --- | --- | --- | --- |
|  | **n=2,429,610** | **n=2,349,353** | **n=80,257** |  |
| **Age** | 56.87 ± 12.42 | 56.59 ± 12.39 | 65.1 ± 10.32 | <0.0001 |
| <40 | 201495 (8.29%) | 200459 (8.53%) | 1036 (1.29%) | <0.0001 |
| 40-64 | 1526041 (62.81%) | 1492146 (63.51%) | 33895(42.23%) |  |
| ≥65 | 702074 (28.9%) | 656748 (27.95%) | 45326 (56.48%) |  |
| **Male sex** | 1458071 (60.01%) | 1409973 (60.02%) | 48098 (59.93%) | 0.6272 |
| **Smoking** |  |  |  | <0.0001 |
| Never | 1346503 (55.42%) | 1299294 (55.3%) | 47209 (58.82%) |  |
| Former | 435772 (17.94%) | 419648 (17.86%) | 16124 (20.09%) |  |
| Current | 647335 (26.64%) | 630411 (26.8%3) | 16924 (21.09%) |  |
| **Drinking** |  |  |  | <0.0001 |
| Non- | 1381064 (56.84%) | 1330298 (56.62%) | 50766 (63.25%) |  |
| Mild | 800591 (32.95%) | 778751 (33.15%) | 21840 (27.21%) |  |
| Heavy | 247955 (10.21%) | 240304 (10.23%) | 7651 (9.53%) |  |
| **Regular exercise** | 498783 (20.53%) | 482350 (20.53%) | 16433 (20.48%) | 0.7008 |
| **Hypertension** | 1336685 (55.02%) | 1278625 (54.42%) | 58060 (72.34%) | <0.0001 |
| **Dyslipidemia** | 967768 (39.83%) | 933892 (39.75%) | 33876 (42.21%) | <0.0001 |
| **BMI, kg/m2** |  |  |  | <0.0001 |
| BMI < 18.5 | 38367 (1.58%) | 36958 (1.57%) | 1409 (1.76%) |  |
| 18.5 ≤ BMI < 23 | 606466 (24.96%) | 587290 (25%) | 19176 (23.89%) |  |
| 23 ≤ BMI < 25 | 603912 (24.86%) | 584562(24.88) | 19350(24.11) |  |
| 25 ≤ BMI < 30 | 995787(40.99) | 962135 (40.95%) | 33652 (41.93%) |  |
| 30 ≤ BMI | 185078 (7.62%) | 178408 (7.59%) | 6670 (8.31%) |  |
| **Waist circumference, cm** | 85.34 ± 8.93 | 85.28 ± 8.89 | 87.02 ± 9.84 | <0.0001 |
| **SBP, mmHg** | 128.97 ± 15.89 | 128.89 ± 15.85 | 131.52 ± 16.85 | <0.0001 |
| **DBP, mmHg** | 79.09 ± 10.29 | 79.08 ± 10.28 | 79.17 ± 10.71 | 0.0204 |
| **Glucose, mg/dL** | 138.06 ± 48.08 | 138.12 ± 48.07 | 136.32 ± 48.39 | <0.0001 |
| **Total cholesterol, mg/dL** | 197.38 ± 46.05 | 197.58 ± 46.05 | 191.45 ± 45.69 | <0.0001 |
| **HDL, mg/dL** | 52.47 ± 32.01 | 52.49 ± 32.12 | 51.88 ± 28.7 | <0.0001 |
| **LDL, mg/dL** | 113.66 ± 91.74 | 113.79 ± 91.8 | 109.88 ± 89.93 | <0.0001 |
| **Diabetes mellitus** |  |  |  |  |
| Disease duration  ≥ 5 year | 1328261 (54.67%) | 1276514 (54.33%) | 51747 (64.48%) | <0.0001 |
| DM medication | 337362 (13.89%) | 324102 (13.8%) | 13260 (16.52%) | <0.0001 |
| ≥3 DM medication | 731987 (30.13%) | 700437 (29.81%) | 31550 (39.31%) | <0.0001 |

**Supplementary Table 3. Subgroup analyses**

|  | **Subgroup** | **Medical Aid Burden** | **No. of participants** | **AF** | **IR  (per 1000)** | **HR (95% CI)** | ***P*-value** | ***P* for interaction** |
| --- | --- | --- | --- | --- | --- | --- | --- | --- |
| **Age** | **<40** | 0 | 199,126 | 1,024 | 0.67 | 1 (Reference) | 0.4403 | <.0001 |
|  |  | 1 | 705 | 3 | 0.58 | 1.06 (0.34,3.29) |  |  |
|  |  | 2 | 545 | 4 | 1.01 | 1.92 (0.72,5.14) |  |  |
|  |  | 3 | 417 | 4 | 1.32 | 2.44 (0.91,6.54) |  |  |
|  |  | 4 | 376 | 0 | 0 | - |  |  |
|  |  | 5 | 326 | 1 | 0.52 | 0.87 (0.12,6.19) |  |  |
|  | **40-65** | 0 | 1,486,221 | 32,801 | 9.00 | 1 (Reference) | <.0001 |  |
|  |  | 1 | 6,042 | 176 | 4.34 | 1.46 (1.25,1.69) |  |  |
|  |  | 2 | 6,091 | 189 | 4.75 | 1.57 (1.36,1.82) |  |  |
|  |  | 3 | 7,022 | 181 | 4.07 | 1.29 (1.12,1.50) |  |  |
|  |  | 4 | 6,049 | 170 | 4.51 | 1.48 (1.28,1.73) |  |  |
|  |  | 5 | 14,616 | 378 | 4.94 | 1.59 (1.44,1.77) |  |  |
|  | **≥65** | 0 | 679,445 | 43,870 | 9.60 | 1 (Reference) | <.0001 |  |
|  |  | 1 | 3,950 | 277 | 11.26 | 1.22 (1.08,1.37) |  |  |
|  |  | 2 | 4,369 | 302 | 11.51 | 1.20 (1.07,1.34) |  |  |
|  |  | 3 | 4,992 | 341 | 11.18 | 1.17 (1.05,1.30) |  |  |
|  |  | 4 | 4,264 | 282 | 10.80 | 1.16 (1.03,1.30) |  |  |
|  |  | 5 | 5,054 | 254 | 8.19 | 1.20 (1.06,1.36) |  |  |
| **Sex** | **Male** | 0 | 1,429,887 | 46,959 | 4.56 | 1 (Reference) | <.0001 | 0.7508 |
|  |  | 1 | 4,938 | 208 | 6.65 | 1.32 (1.15,1.51) |  |  |
|  |  | 2 | 4,642 | 221 | 7.75 | 1.43 (1.25,1.63) |  |  |
|  |  | 3 | 5,068 | 209 | 6.85 | 1.22 (1.06,1.39) |  |  |
|  |  | 4 | 4,341 | 182 | 7.14 | 1.28 (1.11,1.49) |  |  |
|  |  | 5 | 9,195 | 319 | 6.66 | 1.49 (1.33,1.66) |  |  |
|  | **Female** | 0 | 934,905 | 30,736 | 4.55 | 1 (Reference) | <.0001 |  |
|  |  | 1 | 5,759 | 248 | 6.35 | 1.28 (1.13,1.45) |  |  |
|  |  | 2 | 6,363 | 274 | 6.60 | 1.23 (1.09,1.39) |  |  |
|  |  | 3 | 7,363 | 317 | 6.67 | 1.20 (1.08,1.35) |  |  |
|  |  | 4 | 6,348 | 270 | 6.60 | 1.24 (1.10,1.40) |  |  |
|  |  | 5 | 10,801 | 314 | 5.10 | 1.44 (1.28,1.61) |  |  |
| **DM duration** | **<5Y** | 0 | 1,658,344 | 47,231 | 3.88 | 1 (Reference) | <.0001 | 0.7084 |
|  |  | 1 | 7,175 | 292 | 5.99 | 1.34 (1.20,1.51) |  |  |
|  |  | 2 | 6,865 | 283 | 6.17 | 1.28 (1.14,1.44) |  |  |
|  |  | 3 | 7,410 | 301 | 6.25 | 1.25 (1.11,1.40) |  |  |
|  |  | 4 | 6,902 | 274 | 6.18 | 1.21(1.08,1.37) |  |  |
|  |  | 5 | 10,927 | 326 | 5.25 | 1.48 (1.32,1.65) |  |  |
|  | **≥5Y** | 0 | 706,448 | 30,464 | 6.27 | 1 (Reference) | <.0001 |  |
|  |  | 1 | 3,522 | 164 | 7.60 | 1.22 (1.05,1.43) |  |  |
|  |  | 2 | 4,140 | 212 | 8.79 | 1.35 (1.18,1.55) |  |  |
|  |  | 3 | 5,021 | 225 | 7.54 | 1.16 (1.02,1.33) |  |  |
|  |  | 4 | 3,787 | 178 | 8.05 | 1.32 (1.14,1.53) |  |  |
|  |  | 5 | 9,069 | 307 | 6.49 | 1.42 (1.27,1.60) |  |  |
| **Insulin use** | **No** | 0 | 2,185,933 | 68,511 | 4.32 | 1 (Reference) | <.0001 | 0.2233 |
|  |  | 1 | 9,257 | 384 | 6.21 | 1.32 (1.19,1.46) |  |  |
|  |  | 2 | 9,312 | 408 | 6.77 | 1.34 (1.21,1.47) |  |  |
|  |  | 3 | 10,440 | 437 | 6.57 | 1.24 (1.13,1.37) |  |  |
|  |  | 4 | 9,023 | 353 | 6.20 | 1.21 (1.09,1.35) |  |  |
|  |  | 5 | 16,140 | 461 | 5.15 | 1.45 (1.32,1.59) |  |  |
|  | **Yes** | 0 | 178,859 | 9,184 | 7.80 | 1 (Reference) | <.0001 |  |
|  |  | 1 | 1,440 | 72 | 8.47 | 1.18 (0.93,1.49) |  |  |
|  |  | 2 | 1,693 | 87 | 8.95 | 1.20 (0.97,1.48) |  |  |
|  |  | 3 | 1,991 | 89 | 7.76 | 1.04 (0.85,1.29) |  |  |
|  |  | 4 | 1,666 | 99 | 10.42 | 1.41 (1.16,1.73) |  |  |
|  |  | 5 | 3,856 | 172 | 8.67 | 1.42 (1.22,1.67) |  |  |
| **≥3 DM medications** | **No** | 0 | 2,041,715 | 64,982 | 4.39 | 1 (Reference) | <.0001 | 0.2299 |
|  |  | 1 | 8,709 | 378 | 6.57 | 1.36 (1.23,1.51) |  |  |
|  |  | 2 | 8,794 | 391 | 6.93 | 1.34 (1.21,1.48) |  |  |
|  |  | 3 | 9,669 | 410 | 6.73 | 1.24 (1.12,1.36) |  |  |
|  |  | 4 | 8,296 | 358 | 6.91 | 1.30(1.17,1.44) |  |  |
|  |  | 5 | 15,065 | 478 | 5.78 | 1.52 (1.39,1.67) |  |  |
|  | **Yes** | 0 | 323,077 | 12,713 | 5.65 | 1 (Reference) | 0.0051 |  |
|  |  | 1 | 1,988 | 78 | 6.11 | 1.07 (0.85,1.33) |  |  |
|  |  | 2 | 2,211 | 104 | 7.65 | 1.25 (1.03,1.51) |  |  |
|  |  | 3 | 2,762 | 116 | 6.77 | 1.13 (0.94,1.36) |  |  |
|  |  | 4 | 2,393 | 94 | 6.42 | 1.11 (0.91,1.36) |  |  |
|  |  | 5 | 4,931 | 155 | 5.78 | 1.30 (1.11,1.53) |  |  |
| **CKD** | **No** | 0 | 2100256 | 61832 | 4.06 | 1 (Reference) | <.0001 | 0.0146 |
|  |  | 1 | 8959 | 343 | 5.73 | 1.33 (1.19,1.48) |  |  |
|  |  | 2 | 9048 | 337 | 5.73 | 1.24 (1.11,1.38) |  |  |
|  |  | 3 | 10219 | 362 | 5.55 | 1.16 (1.04,1.28) |  |  |
|  |  | 4 | 8825 | 319 | 5.74 | 1.22 (1.09,1.37) |  |  |
|  |  | 5 | 17056 | 462 | 4.92 | 1.41 (1.28,1.54) |  |  |
|  | **Yes** | 0 | 264536 | 15863 | 8.81 | 1 (Reference) | <.0001 |  |
|  |  | 1 | 1738 | 113 | 10.82 | 1.18 (0.98,1.42) |  |  |
|  |  | 2 | 1957 | 158 | 14.10 | 1.46 (1.25,1.71) |  |  |
|  |  | 3 | 2212 | 164 | 12.78 | 1.30 (1.12,1.52) |  |  |
|  |  | 4 | 1864 | 133 | 12.31 | 1.32 (1.11,1.56) |  |  |
|  |  | 5 | 2940 | 171 | 10.95 | 1.56 (1.34,1.82) |  |  |
| **Hypertension** | **No** | 0 | 1069644 | 21598 | 2.74 | 1 (Reference) | <.0001 | 0.2158 |
|  |  | 1 | 4195 | 113 | 3.95 | 1.38 (1.15,1.67) |  |  |
|  |  | 2 | 3972 | 102 | 3.87 | 1.30 (1.07,1.58) |  |  |
|  |  | 3 | 4247 | 114 | 4.14 | 1.32 (1.10,1.59) |  |  |
|  |  | 4 | 3719 | 110 | 4.66 | 1.48 (1.23,1.79) |  |  |
|  |  | 5 | 7148 | 160 | 4.07 | 1.71 (1.46,2.00) |  |  |
|  | **Yes** | 0 | 1295148 | 56097 | 6.13 | 1 (Reference) | <.0001 |  |
|  |  | 1 | 6502 | 343 | 8.22 | 1.28 (1.15,1.42) |  |  |
|  |  | 2 | 7033 | 393 | 9.00 | 1.32 (1.20,1.46) |  |  |
|  |  | 3 | 8184 | 412 | 8.16 | 1.19 (1.07,1.31) |  |  |
|  |  | 4 | 6970 | 342 | 7.99 | 1.20 (1.08,1.34) |  |  |
|  |  | 5 | 12848 | 473 | 6.75 | 1.39 (1.27,1.52) |  |  |
| **Dyslipidemia** | **No** | 0 | 1429089 | 45089 | 4.36 | 1 (Reference) | <.0001 | 0.1287 |
|  |  | 1 | 5977 | 252 | 6.42 | 1.33 (1.17,1.50) |  |  |
|  |  | 2 | 5924 | 237 | 6.27 | 1.20 (1.05,1.36) |  |  |
|  |  | 3 | 6451 | 275 | 6.77 | 1.24 (1.10,1.39) |  |  |
|  |  | 4 | 5383 | 235 | 7.08 | 1.32 (1.16,1.50) |  |  |
|  |  | 5 | 9018 | 293 | 5.97 | 1.59 (1.42,1.79) |  |  |
|  | **Yes** | 0 | 935703 | 32606 | 4.87 | 1 (Reference) | <.0001 |  |
|  |  | 1 | 4720 | 204 | 6.57 | 1.27 (1.11,1.46) |  |  |
|  |  | 2 | 5081 | 258 | 8.01 | 1.45 (1.29,1.64) |  |  |
|  |  | 3 | 5980 | 251 | 6.71 | 1.19 (1.05,1.35) |  |  |
|  |  | 4 | 5306 | 217 | 6.53 | 1.21 (1.05,1.38) |  |  |
|  |  | 5 | 10978 | 340 | 5.63 | 1.39 (1.24,1.55) |  |  |

**Supplementary Figure 1. Income level and the risk of atrial fibrillation**

**
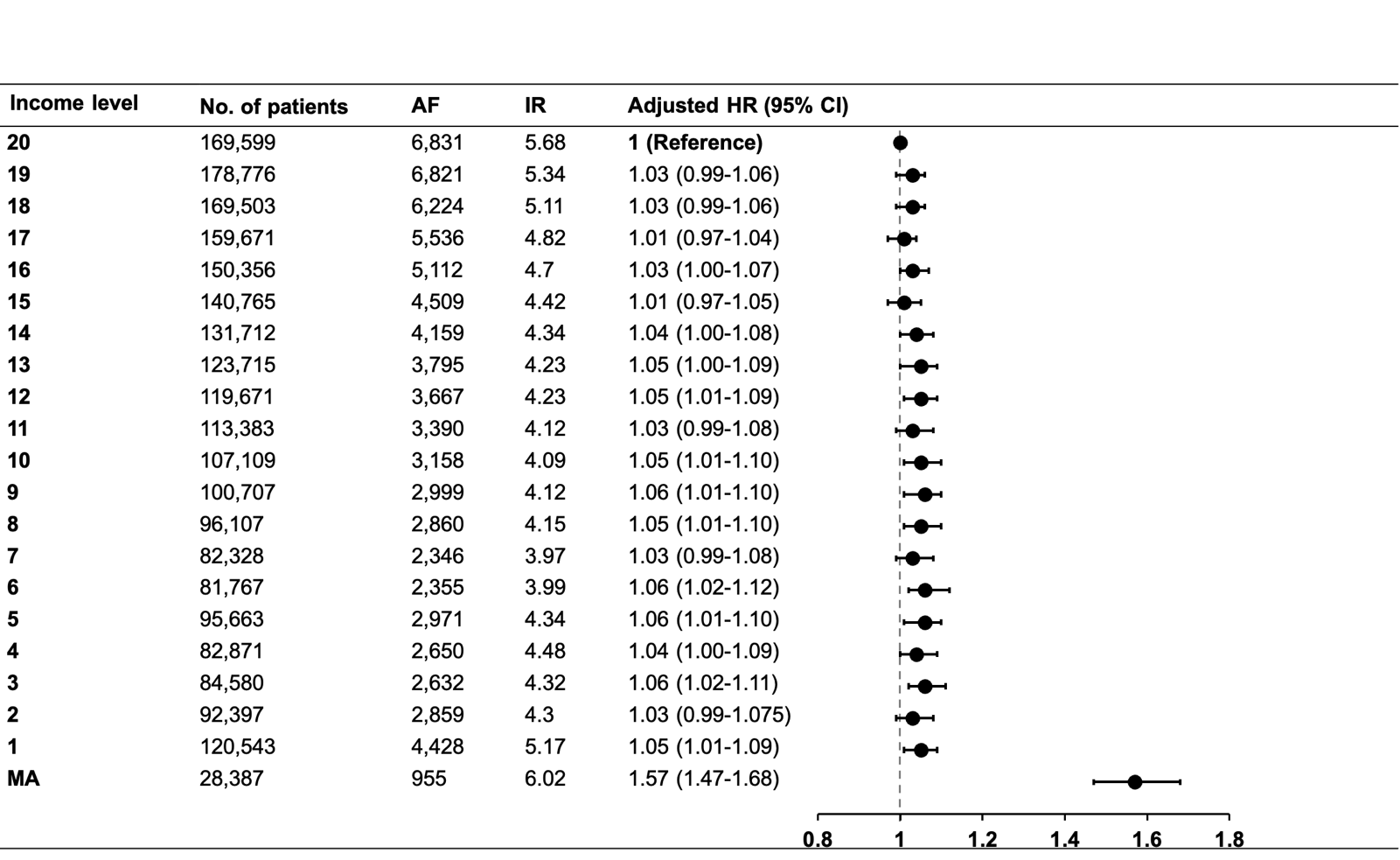
**

**Supplementary Figure 2. Subgroup analyses**


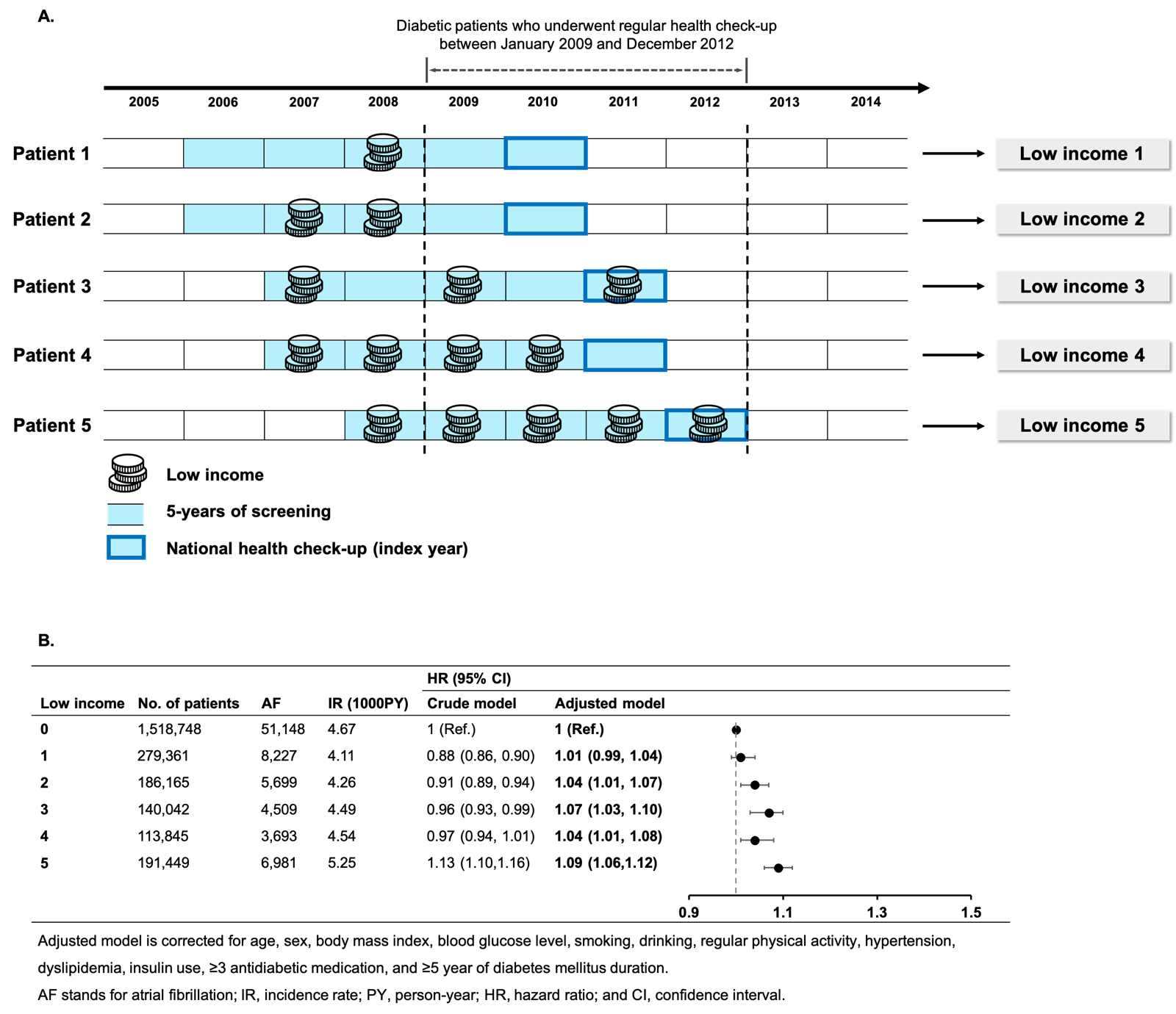

Supplement: Supplementary file 1 [file Data_Sheet_1.DOCX]
